# Distinct histopathological features are associated with molecular subtypes and outcome in low grade serous ovarian carcinoma

**DOI:** 10.1038/s41598-023-34627-5

**Published:** 2023-05-11

**Authors:** Robert L. Hollis, John P. Thomson, Juliette van Baal, Narthana Ilenkovan, Michael Churchman, Koen van de Vijver, Frederike Dijk, Alison M. Meynert, Clare Bartos, Tzyvia Rye, Ian Croy, Patricia Diana, Mignon van Gent, Helen Creedon, Rachel Nirsimloo, Christianne Lok, Charlie Gourley, C. Simon Herrington

**Affiliations:** 1 Nicola Murray Centre for Ovarian Cancer Research, Cancer Research UK Scotland Centre, Institute of Genetics and Cancer, University of Edinburgh, Crewe Road South, Edinburgh, EH4 2XU UK; 2 Department of Gynaecologic Oncology, The Netherlands Cancer Institute, Antoni Van Leeuwenhoek, Amsterdam, The Netherlands; 3 Department of Pathology, The Netherlands Cancer Institute, Antoni Van Leeuwenhoek, Amsterdam, The Netherlands; 4 Cancer Research UK Scotland Centre, Beatson Institute for Cancer Research, Glasgow, UK; 5 Department of Gynaecologic Oncology, Amsterdam University Medical Centres, Amsterdam, The Netherlands; 6 Department of Pathology, Amsterdam University Medical Centres, Amsterdam, The Netherlands; 7 MRC Human Genetics Unit, Institute of Genetics and Cancer, University of Edinburgh, Edinburgh, UK; 8 Edinburgh Cancer Centre, Western General Hospital, NHS Lothian, Edinburgh, UK

**Keywords:** Ovarian cancer, Cancer genomics, Tumour biomarkers, Cancer

## Abstract

Low grade serous ovarian carcinoma (LGSOC) demonstrates unique clinical and molecular features compared to other ovarian cancer types. The relationship between common histological features of LGSOC and molecular events, such as hormone receptor expression patterns and MAPK gene mutation status, remains poorly understood. Recent data suggest some of these molecular features may be biomarkers of response to recently introduced biologically-targeted therapies, namely endocrine therapy and MEK inhibitors. We utilize a cohort of 63 pathologically-confirmed LGSOC cases with whole exome sequencing and hormone receptor expression data to investigate these relationships. LGSOC cases demonstrated uniformly high oestrogen receptor (ER) expression, but variable progesterone receptor (PR) expression intensity. 60% and 37% of cases demonstrated micropapillary and macropapillary patterns of stromal invasion, respectively. 63% of cases demonstrated desmoplasia, which was significantly associated with advanced disease stage and visible residual disease after cytoreductive surgery. MAPK-mutant cases (*KRAS*, *BRAF*, *NRAS*) more frequently demonstrated macropapillary stromal invasion, while Chr1p loss was associated with desmoplasia and low PR expression. Presence of micropapillary stromal invasion and low PR expression were associated with significantly poorer survival after accounting for stage and residual disease status. Together, these data identify novel relationships between histopathological features and molecularly-defined subgroups in LGSOC.

## Introduction

Low grade serous ovarian carcinoma (LGSOC) is an uncommon ovarian cancer (OC) type, accounting for around 5% of diagnoses^1,2^. LGSOC is now recognised to be a unique disease entity, with distinct clinical behaviour and markedly different molecular profile compared to more common high grade serous ovarian carcinomas (HGSOC, 70% of cases)^1,2^.

Molecularly, LGSOC demonstrates recurrent MAPK pathway-activating mutations in *KRAS*, *NRAS* and *BRAF* (in 33%, 10% and 10% of cases, respectively)^3–8^, which occur mutually exclusively with one another. LGSOC is overwhelmingly *TP53* wild-type^3–7^ and demonstrates relative genomic stability with low tumour mutation burden and few copy number (CN) aberrations^6,7^. By contrast, HGSOC is universally *TP53* mutant, demonstrates high levels of genomic instability and does not harbour recurrent *KRAS*, *BRAF* or *NRAS* mutations^9,10^. The vast majority of LGSOC demonstrate expression of oestrogen receptor (ER) and many express progesterone receptor (PR)^8,11,12^.

Clinically, LGSOC is characterised by young patient age at diagnosis (median approximately 48 years)^3,4^, frequent advanced stage at diagnosis (≥ 80% FIGO stage III-IV)^1,3,5,13^ and high levels of intrinsic chemoresistance (first-line response rate to platinum-based chemotherapy ≤ 25%)^14,15^. By comparison, HGSOC occurs at an older age (median 60–61 years) and is initially highly chemosensitive (first-line response rate ~ 80%)^14,16–18^. LGSOC is also characterised by prolonged post-relapse survival. Recent advances in OC treatment have led to incorporation of endocrine agents and MEK inhibitors into LGSOC management^19^.

Multiple studies have demonstrated a survival benefit for LGSOC patients undergoing treatment with hormone therapy such as letrozole or tamoxifen^20–23^, and current guidelines recommend the use of these drugs as maintenance regimens for LGSOC treatment^19^. The extent of hormone receptor expression has been correlated with clinical benefit from endocrine therapies in OC^24,25^, though this relationship has not been demonstrated in LGSOC specifically. Very recently, treatment with the MEK inhibitor trametinib has been shown to improve PFS in recurrent or persistent LGSOC compared to physician’s choice standard of care^26^; trametinib and binimetinib, another MEK inhibitor, are now recommended for use in the recurrent disease setting^19^. Canonical MAPK gene mutations (*KRAS*, *BRAF*, *NRAS*) have been associated with improved response rate to MEK inhibition^26,27^.

At the histopathological level, LGSOC demonstrates a number of common features. LGSOC is often associated with serous borderline ovarian tumours, the recognised precursor lesion of this tumour type^28^, and around 60% of cases have a borderline component. In contrast to serous borderline tumours, which by definition do not demonstrate stromal invasion, LGSOC demonstrates definitive invasion. Stromal invasion with papillary structures is common; these may be macropapillary (MaP), where invasive papillary structures contain fibrovascular cores^29^, or micropapillary (MiP), where fibrovascular cores are absent^30^. MiP has been associated with poor prognosis for malignancies across a number of anatomical sites, including the cervix^31^, breast^32^, bladder^33^ and lung^34^. The presence of reactive fibrotic stroma, commonly referred to as desmoplasia, is also frequently identified in LGSOC^35^. Desmoplasia is a common feature of multiple tumour types and has been associated with poor prognosis in some settings, including pancreatic cancer^36^.

While recent studies have advanced our understanding of the molecular and clinical behaviour of LGSOC, the relationship between common histopathological features, recurrent molecular events, and clinical behaviour remains understudied. Specifically, it is unclear if certain histological features are indicative of specific molecular events, such as MAPK-activating mutations or hormone receptor expression patterns. Given the biological rationale and emerging data supporting association between specific molecular events and response to regimens such as endocrine therapy and MEK inhibition^24–27^, identifying histopathological features of molecular LGSOC subtypes may be extremely valuable.

## Results

### Histopathological features of LGSOC

Clinical characteristics of the 63 pathologically confirmed LGSOC cases are outlined in Table 1. Pure borderline tumours were specifically excluded; all cases demonstrated definitive stromal invasion. The median progression-free and disease-specific survival times of the LGSOC cohort were 5.5 and 12.9 years respectively.

**Table 1:** Characteristics of low grade serous ovarian carcinoma cases.

		N	%
Cases	Total	63	–
Age	Median (years)	54	Range 19–66
FIGO stage	I–II	14	22.6
	III	39	62.9
	IV	9	14.5
	NA	1	–
RD after debulking surgery	Macroscopic	34	55.7
	No visible RD	27	44.3
	Unknown	2	–
First-line management	Adjuvant, single-agent platinum	7	11.1
	Adjuvant, platinum-taxane combination	24	38.1
	Adjuvant, other platinum combination	1	1.6
	Neoadjuvant platinum-taxane	14	22.2
	Neoadjuvant, other platinum combination	1	1.6
	Surgery only	15	23.8
	Letrozole only	1	1.6
Macropapillary invasion pattern	No	40	63.5
	Yes	23	36.5
Micropapillary invasion pattern	No	25	39.7
	Yes	38	60.3
Desmoplasia	No	23	36.5
	Yes	40	63.5
Diagnosis period	Pre-2000	10	15.9
	2000s	24	38.1
	2010 onward	29	46.0
Death details at last follow-up	Alive	25	39.7
	Died of OC	21	33.3
	Died, non-OC or unknown cause	17	27.0

RD, residual disease; OC, ovarian carcinoma.

**Figure 1:**
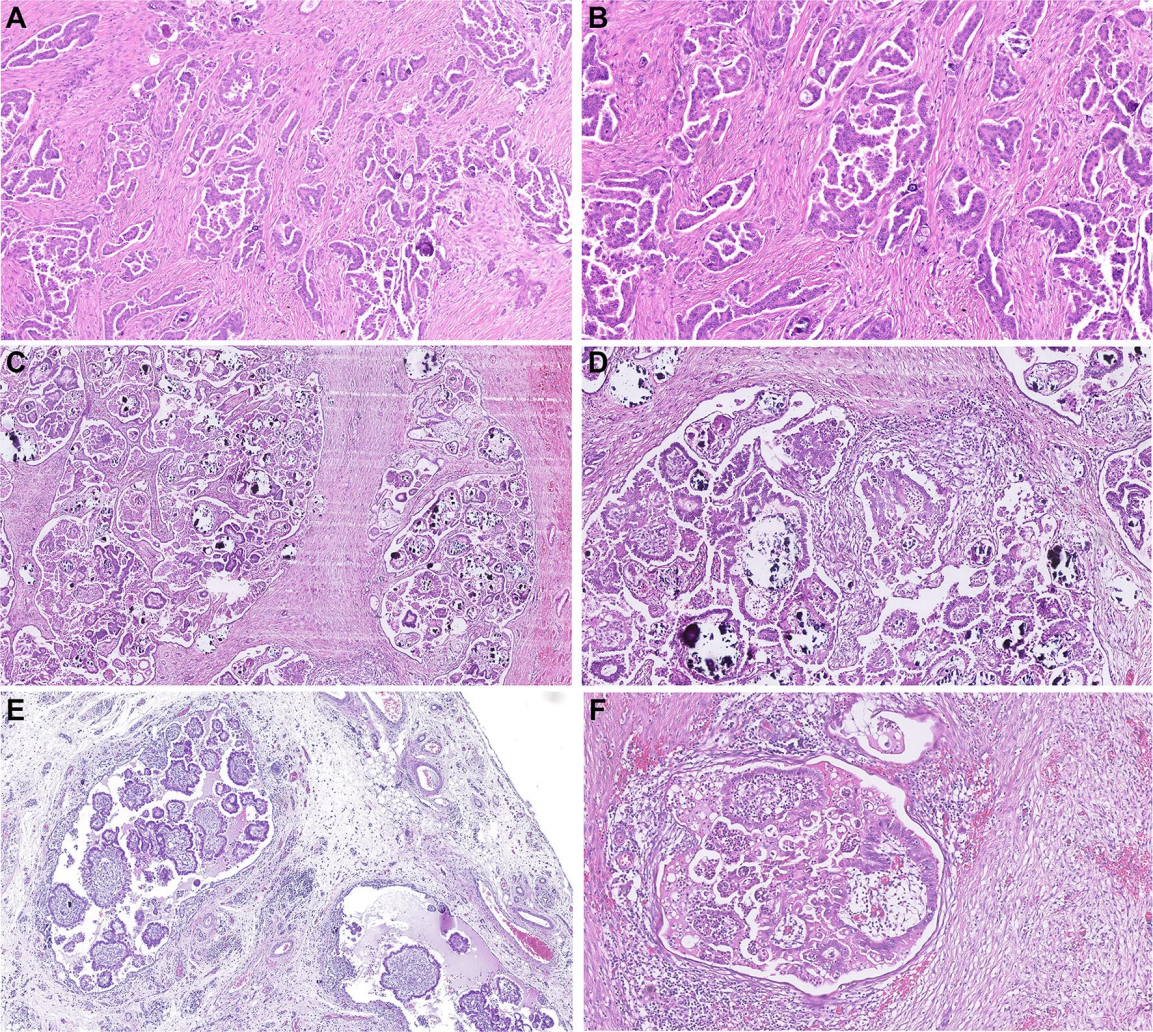
(**A**,**B**) Micropapillary (MiP) invasion, in which the tumour invades the stroma as small papillary structures composed only of tumour cells. The tumour clusters may form glandular spaces, as seen in the images, but there are no fibrovascular cores. (**C**,**D**) Macropapillary (MaP) invasion, in which the infiltrating tumour forms papillary structures that contain fibrovascular cores. (**E**) An example in which the MaP invasion pattern is maintained in an omental metastasis. (**F**) MaP invasion (left) associated with stromal desmoplasia (right).

**Figure 2:**
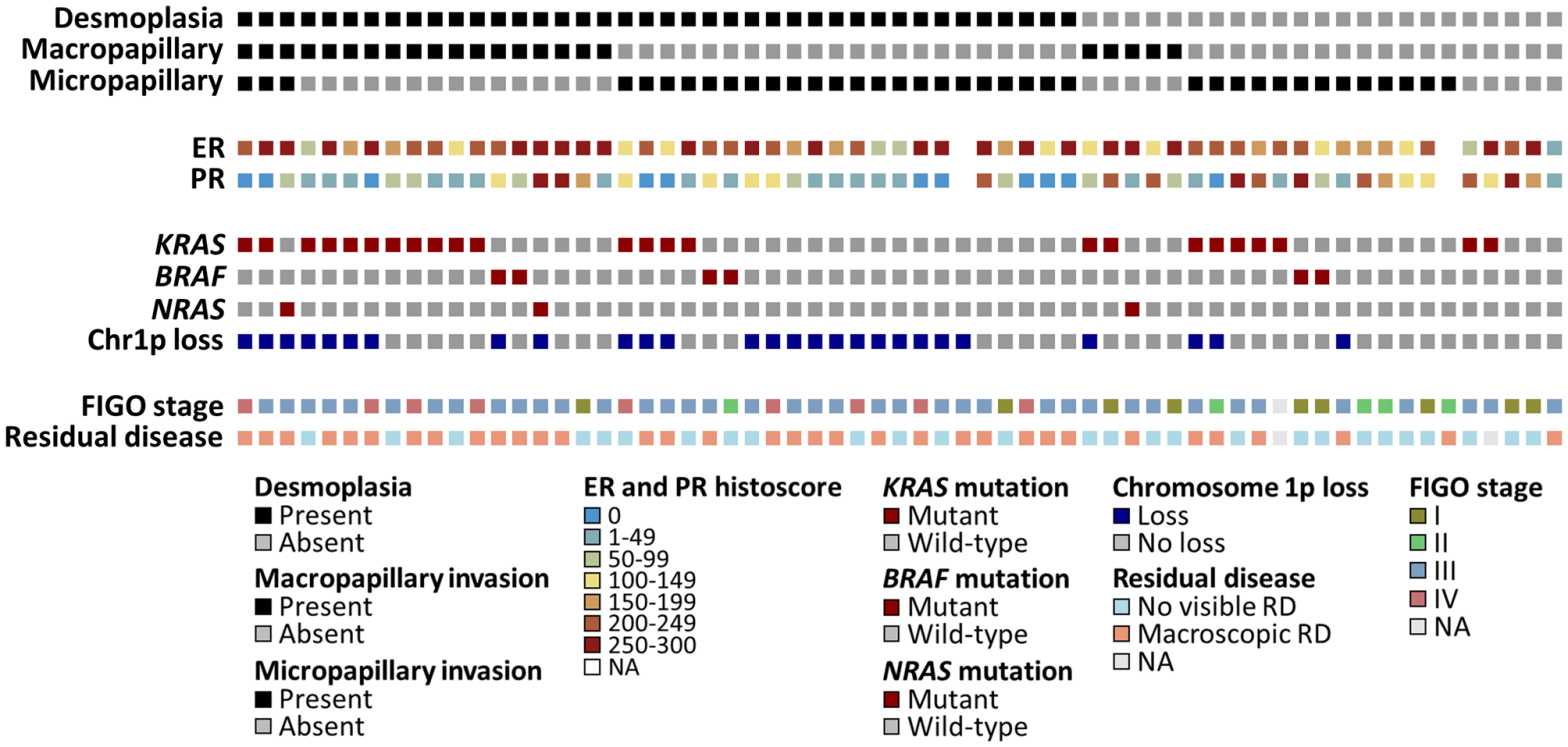
Histopathological, molecular and clinical characteristics of low grade serous ovarian carcinoma. ER, oestrogen receptor. PR, progesterone receptor. RD, residual disease. NA, not available.

The micropapillary (MiP) pattern of invasion was identified in 38 cases (60%) (Figs. 1A,B, 2), while the macropapillary (MaP) invasion pattern was identified in 23 cases (37%) (Fig. 1C–F). The MiP and MaP patterns were largely mutually exclusive (P < 0.001, co-occurrence in only 3 cases) (Fig. 2). 5 cases demonstrated neither MaP nor MiP patterns, but showed glandular, cribriform or solid patterns of invasion.

**Figure 3:**
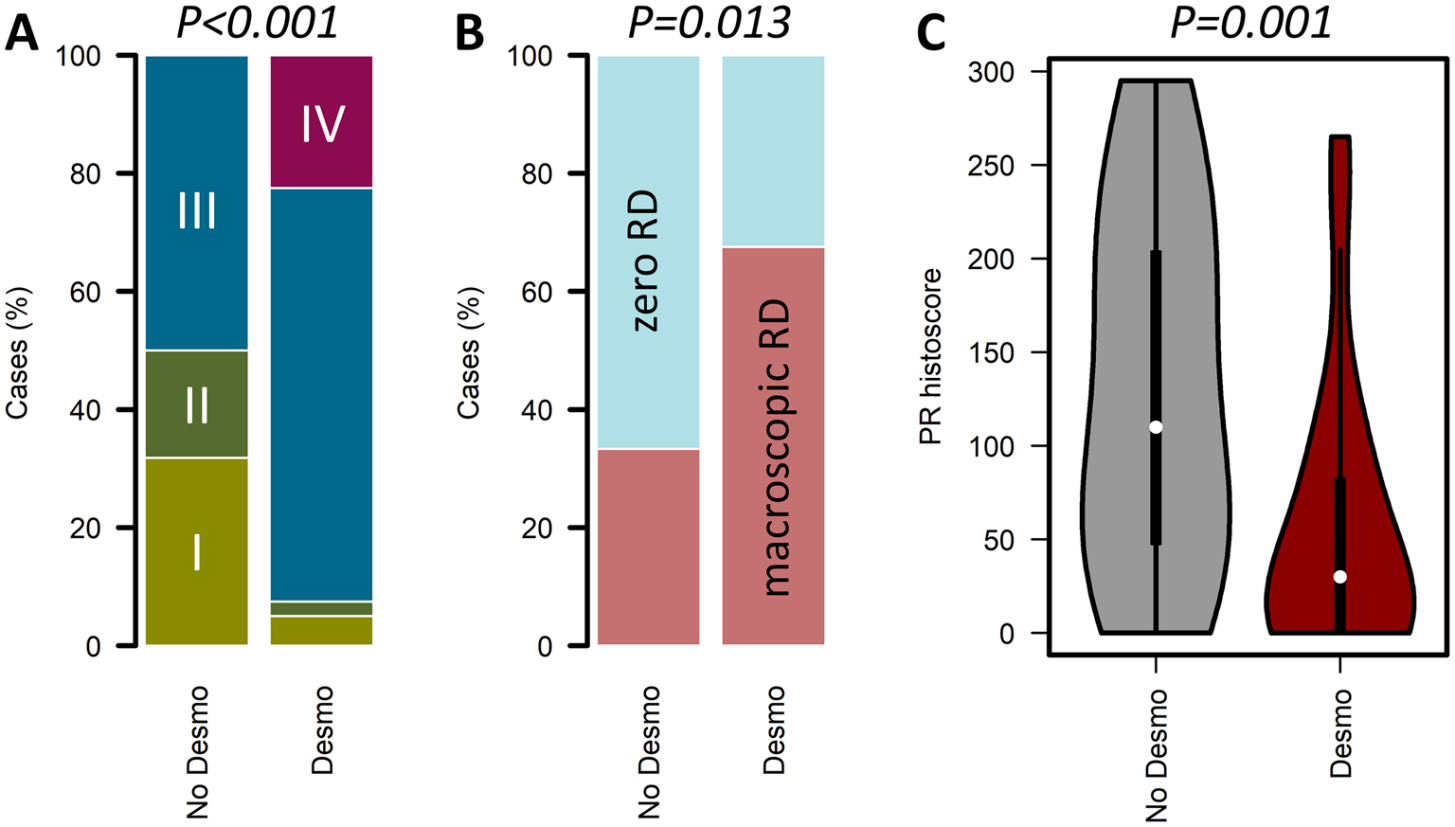
Features associated with desmoplasia in low grade serous ovarian carcinoma. (**A**) Distribution of stage at diagnosis between tumours with desmoplasia (n = 40: 2 FIGO stage I, 1 stage II, 28 stage III, 9 stage IV) and without desmoplasia (n = 23: 7 stage I, 4 stage II, 11 stage III, 1 not evaluable). (**B**) Distribution of residual disease (RD) between tumours with desmoplasia (n = 40: 27 macroscopic RD, 13 zero visible RD) and without desmoplasia (n = 23: 7 macroscopic RD, 14 zero visible RD, 2 not evaluable). (**C**) Progesterone receptor (PR) expression between cases with and without desmoplasia (median PR histoscore 30 and 110, respectively). Desmo, desmoplastic; No des, no desmoplasia identified; zero RD, zero macroscopic residual disease after debulking surgery; macro RD, macroscopic residual disease after debulking surgery.

Desmoplasia was identified in 40 cases (63%) (Figs. 1F, 2). Desmoplasia was significantly associated with advanced stage at diagnosis (93%, 37 of 40 desmoplastic cases with FIGO III/IV vs 50%, 11 of 22 evaluable non-desmoplastic cases with FIGO III/IV; P < 0.001) (Fig. 3A) and presence of macroscopic residual disease (RD) after debulking surgery (68%, 27 of 40 desmoplastic cases demonstrated macroscopic RD vs 33%, 7 of 21 evaluable non-desmoplastic cases, P = 0.013) (Fig. 3B).

### ER and PR expression patterns

LGSOC samples uniformly expressed high levels of ER (median histoscore 220; 98%, 60 of 61 evaluable cases demonstrate ER histoscore ≥ 50) (Supplementary Fig.  S1, Supplementary Fig. S2A). The median PR histoscore was 50 (range 0–295) (Supplementary Fig. S2B).

Desmoplastic tumours demonstrated significantly lower PR expression (median histoscore 30 vs 110, P = 0.001) (Fig. 3C) than non-desmoplastic tumours. There was no significant difference in PR expression between tumours based on presence of MaP or MiP invasion patterns.

### Impact of features on LGSOC outcome

**Figure 4:**
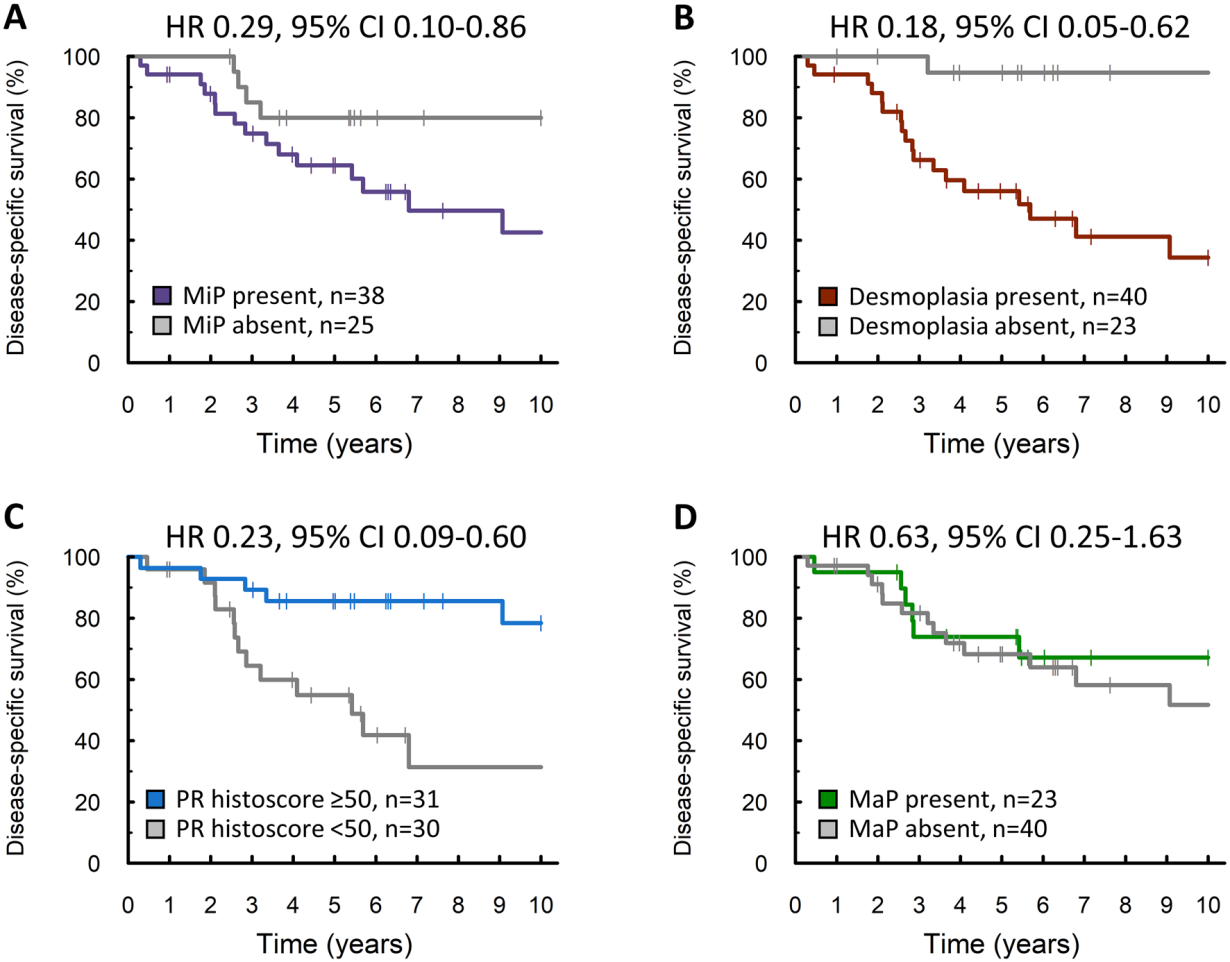
Univariable analysis of disease-specific survival (DSS). (**A**) DSS of cases with and without the micropapillary (MiP) pattern of stromal invasion. Labelled hazard ratio represent comparison of cases without MiP versus cases with MiP stromal invasion (P = 0.025). (**B**) DSS of cases with and without desmoplasia. Labelled hazard ratio represent comparison of cases without desmoplasia identified versus desmoplastic cases (P = 0.006). (**C**) DSS of cases classified by progesterone receptor (PR) expression levels. Labelled hazard ratio represent comparison of cases with PR histoscore ≥ 50 (the median) versus PR histoscore < 50 (P = 0.003) (2 cases not evaluable for PR expression). (**D**) DSS of cases with and without the macropapillary (MaP) pattern of stromal invasion. Labelled hazard ratio represent comparison of cases with MaP versus cases without MaP stromal invasion (P > 0.2).

Patients with LGSOC demonstrating the MiP invasion pattern experienced significantly shorter disease-specific survival (DSS) (HR for absence of MiP 0.29, 95% CI 0.10–0.86) (Fig. 4A), which remained significant after accounting for stage at diagnosis and RD status (P = 0.029, Supplementary Table S1). Desmoplasia was also associated with shorter DSS (HR for absence of desmoplasia 0.18, 95% CI 0.05–0.62) (Fig. 4B), but this did not remain statistically significant after accounting for stage and RD status (P = 0.054, Supplementary Table S2). Conversely, high PR expression (greater than or equal to the median PR histoscore of 50) was associated with significantly prolonged survival (HR 0.23, 95% CI 0.09–0.60) (Fig. 4C), which remained significant after accounting for stage and RD status (P = 0.036, Supplementary Table S3). MaP invasion was not significantly associated with survival time (Fig. 4D). Analysis of progression-free survival identified similar phenotypes (Supplementary Fig. S3).

### Relationship of histopathological features with genomic events

**Figure 5:**
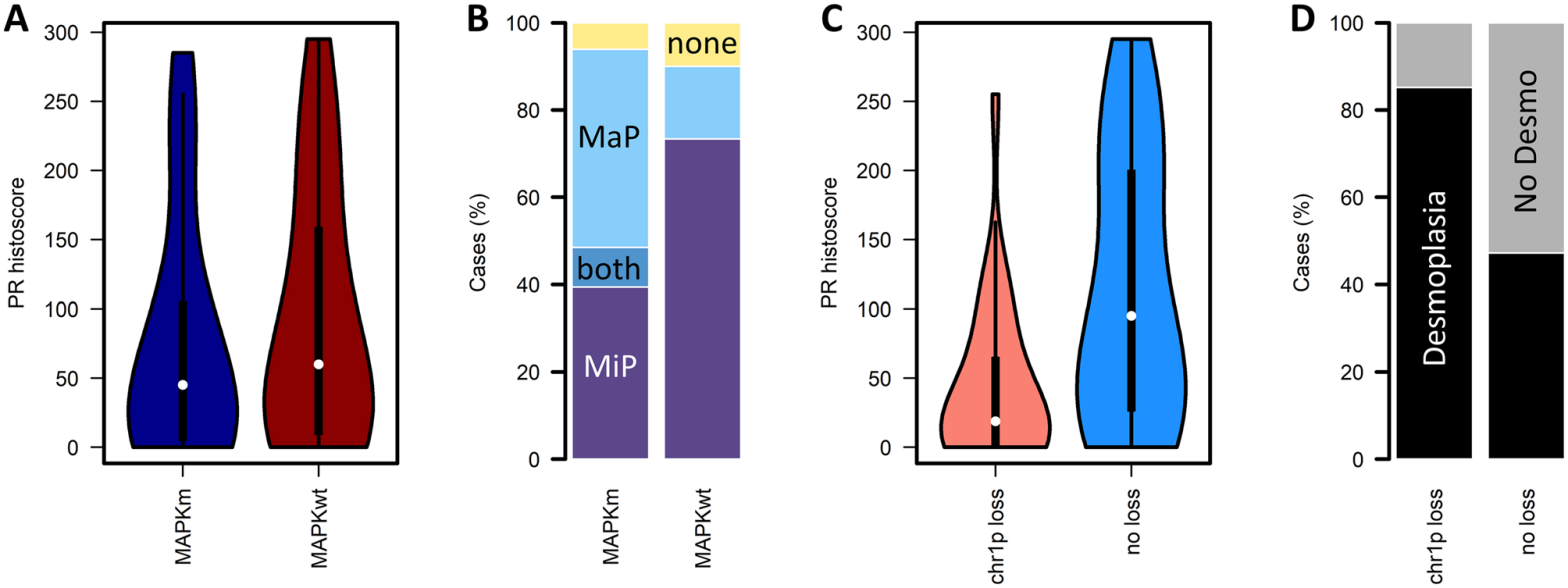
Relationship between histopathological and molecular features in low grade serous ovarian carcinoma (LGSOC). (**A**) Progesterone receptor (PR) expression levels between MAPK-mutant (MAPKm) and MAPK wild-type (MAPKwt) cases. (**B**) Histopathological patterns of invasion across MAPKm and MAPKwt cases. (**C**) PR expression levels according to chr1p loss status. (**D**) Frequency of desmoplasia according to chr1p loss status. MAPKm, gene mutation affecting *KRAS*, *NRAS* or *BRAF*. MAPKwt, wild-type for *KRAS*, *NRAS* and *BRAF*. MaP, macropapillary stromal invasion present. MiP, micropapillary stromal invasion present. Both, both MiP and MaP present. None, neither MiP nor MaP present (solid, cribriform or glandular stromal invasion without MiP or MaP). No desmo, no desmoplasia identified.

Of the 63 cases, 33 (52%) demonstrated mutation of *KRAS*, *BRAF* or *NRAS* (MAPKm group: n = 24 *KRAS*, n = 6 *BRAF*, n = 3 *NRAS*); 30 cases were wild-type for these genes (MAPKwt group). The MaP pattern of stromal invasion was significantly more frequent in MAPKm cases (55%, 18 of 33 cases) compared to the MAPKwt group (27%, 5 of 30 cases; P = 0.002; Fig. 5A). There was no significant difference in PR expression between the MAPKm and MAPKwt cases (median 45 vs 60, P = 0.477) (Fig. 5B).

The most common copy number events were loss of chr1p (43%, 27 cases), loss of 22q (32%, 20 cases), gain of 1q (37%, 23 cases) and gain of 19p (35%, 22 cases). Loss of chr1p was associated with significantly lower PR expression (median histoscore 19 vs 95, P = 0.004, P-adj = 0.016) (Fig. 5C); other recurrent CN events were not associated with differential PR expression after adjustment for multiple testing (Supplementary Fig. S4). Loss of chr1p was significantly associated with presence of desmoplasia (85%, 23 of 27 chr1p loss cases with desmoplasia vs 47%, 17 of 36, in cases without loss; P = 0.002) (Fig. 5D).

## Discussion

LGSOC is a unique form of OC that is typically diagnosed in younger women^3,4^; LGSOC therefore affects a disproportionate number of life years compared to other OC types. LGSOC demonstrates high levels of intrinsic resistance to conventional chemotherapy^14^, and biologically-targeted treatment strategies have therefore become the focus for improving patient survival.

LGSOC demonstrates high levels of hormone receptor expression^8,11,12^, and a high frequency of mutation in core MAPK components^3–8^. Endocrine therapy and MEK inhibitors, targeted at these recurrent molecular features, have recently emerged as effective treatment strategies for LGSOC^20–23,26^. However, response to these targeted therapeutic options is variable in patient populations, and increasing evidence—both from in vitro studies and translational analysis of trial specimens^26,27,37,38^—suggests the tumour’s molecular profile is associated with response and patient benefit. Both the MILO and GOG281 studies of MEK inhibitors for recurrent or persistent LGSOC have suggested improved response in patients whose tumours harbour MAPK pathway mutations^26,27^. MILO suggested improved response rate to binimetinib for those harbouring *KRAS* mutations^27^, while GOG281 suggested improved response rate for cases with *KRAS*, *BRAF* or *NRAS* mutations^26^. Though the precise mechanisms of intrinsic and acquired resistance to both chemotherapy and biological agents remain poorly understood in LGSOC, in vitro data suggest that NOTCH pathway activation may contribute to MEK inhibitor resistance^39^. Other factors that could impact treatment response or survival, such as expression of drug efflux pumps or modulation of the host immune response, remain under-explored.

Biomarkers of response to endocrine therapy in LGSOC are poorly understood. In HGSOC, higher ER expression has been associated with significantly improved response and clinical benefit from endocrine agents^24,25^. However, this has not been demonstrated for LGSOC specifically. ER is uniformly highly expressed in LGSOC^8,11,12^; this limited dynamic range is an obstacle for associating expression intensity with response. However, PR expression is more variable in LGSOC cases^8,11,12^, representing a marker that may facilitate discrimination of responders from non-responders.

We examined the relationship between hormone receptor expression levels, histopathological features and recurrent genomic events in LGSOC, with the aim of identifying whether molecular subgroups of LGSOC demonstrate specific differences at the histopathological level. Given that hormone receptor expression levels and genomic features such as MAPK mutation may represent biomarkers of endocrine therapy and MEK inhibitor response, histopathological features associated with these molecular events are a candidate strategy for future therapeutic stratification that would not require tumour molecular profiling. Histopathological biomarkers that are identifiable from routine H&E-stained slides could be readily integrated into current diagnostic pathology pipelines. Moreover, the association of histopathological features with patient survival may provide useful prognostic information in LGSOC.

Our study suggests specific associations between histopathological and molecular features. MaP invasion is associated with MAPKm, while desmoplasia is associated with loss of chr1p and low expression of PR. Upon univariable analysis, desmoplasia was associated with poor outcome; however, desmoplasia was strongly associated with advanced stage disease and RD status. The association of desmoplasia with survival did not cross the threshold for significance in multivariable analysis accounting for stage and RD status (P = 0.054). While the MiP invasion pattern was not significantly associated with specific molecular features, patients with tumours demonstrating MiP experienced significantly poorer survival. This is consistent with findings across multiple tumour sites associating MiP with aggressive disease and poorer patient outcomes^31–34^.

Conversely, high expression of PR was associated with significantly improved survival. This is consistent with studies quantifying PR expression by Allred scoring^12^ in LGSOC, and with data in other OC types showing favourable prognosis for cases with high PR expression^11^. In endometrioid OC, high PR expression has been associated with excellent survival^40^, and it has been suggested that de-escalation of adjuvant chemotherapy to endocrine therapy may be feasible for PR-high patients who present with early stage disease^41^. Given the favourable survival of LGSOC demonstrating high PR expression, and the low baseline response rate of LGSOC to chemotherapy^14,15^, this approach may also be feasible in the context of LGSOC.

Optimal tumour debulking, achieving zero macroscopic RD, is a key factor associated with prolonged survival across OC types^42–46^. We show that desmoplasia is associated with lower rates of complete resection in LGSOC, contributing to the poor outcomes experienced by this patient group. Optimizing debulking strategies, improving surgical techniques and intra-operative disease mapping, and focussing radical debulking efforts remain high priorities for improving OC survival^47,48^. Such improvements may be expected to provide particular benefit for poor prognosis patient groups that experience lower rates of complete debulking with current techniques, such as desmoplastic LGSOC.

The core strength of our study is the integration of histopathological data with quantified hormone receptor expression and genomic data derived from whole exome sequencing. Specific exclusion of borderline tumours, with this study only investigating LGSOC with definitive stromal invasion, nuclear WT1 expression and wild-type p53 immunoprofile, is also a major strength, alongside the extensive follow-up period (median 13 years). However, the stringency of inclusion criteria limited the total number of cases (n = 63). This inevitably restricted the statistical power of comparisons between groups, which is the major limitation of our study, though our cohort remains larger than many previous LGSOC studies to date^6,7,49^, and on par with many contemporary studies in this tumour type^5,12^. Our study cohort were also treated in the era prior to routine use of MEK inhibitors and endocrine therapy for LGSOC; however, this limitation does not detract from the associations we describe between histopathological features and molecular events. Moreover, given the recency of updated guidelines to include these therapies as standard of care^19^, investigations of LGSOC patient cohorts treated within these contemporary guidelines would themselves be significantly limited by short follow-up times.

In conclusion, we demonstrate that specific histopathological features are associated with molecular events and outcome in LGSOC. Tumours demonstrating the MaP pattern of stromal invasion more frequently harboured core MAPK pathway mutations (*KRAS*, *BRAF*, *NRAS*). LGSOC demonstrating MaP invasion may therefore represent cases more likely to respond to MEK inhibitors. Conversely, desmoplastic LGSOC, which frequently harbour chr1p loss, are associated with low PR expression; investigation of whether this patient group derive less benefit from endocrine therapy may be warranted. MiP invasion and low PR expression were associated with poor prognosis, independent of stage at diagnosis and extent of RD after debulking surgery.

## Methods

### Patient cohort and ethics

The study cohort comprised 63 cases with pathologically confirmed LGSOC demonstrating definitive stromal invasion following contemporary pathology review by two expert gynaecological pathologists (CSH, KvdV) as part of a previous genomic profiling study^50^. Of 256 potential cases with a diagnosis of LGSOC, serous grade I ovarian carcinoma or serous borderline ovarian tumour identified at the Edinburgh Cancer Centre, Amsterdam University Medical Centres and The Netherland Cancer Institute, 204 cases had material available for pathology review. 118 cases met the inclusion criteria of LGSOC histology with definitive stromal invasion, WT1 positivity and wild-type p53 immunoprofile. Pure borderline tumours were specifically excluded. 44 cases were excluded during quality control prior to genomic characterisation (insufficient tumour cellularity or material for DNA extraction, insufficient DNA yield for WES), and 11 failed sequencing quality control. For full details, see ref^50,51^.

The study was registered with and received ethical approval from the Lothian Human Annotated Bioresource (#15/ES/0094/SR925), NKI-AVL Translational Research Board (#CFMPB284), and University of Amsterdam AMC Biobank Assessment Committee (2016_070#A201641). All relevant ethical regulations have been complied with, including the need for written informed from all cases or their legal representatives; all experiments were performed in accordance with relevant guidelines and regulations. Reporting of study findings were informed by the EQUATOR network principles, but did not conform strictly to CONSORT reporting guidelines due to the non-interventional nature of the study.

### Clinical annotation

Baseline characteristics, treatment and outcome data were collected from the Edinburgh Ovarian Cancer Database and from patient file review. Recurrence and progression events were defined using radiological investigation, GCIG CA125 tumour marker criteria, pathologically-confirmed recurrence or disease-specific death events. The median follow-up time was 13.3 years, as determined by the reverse Kaplan–Meier method.

### Quantification of ER and PR expression

Immunohistochemistry for ER and PR was performed using protocol F on the Leica BOND III Autostainer with epitope retrieval solution 2 for 20 min. ER immunohistochemistry used rabbit anti-ER antibody M3643 clone EP1; PR immunohistochemistry used mouse anti-PR antibody M3569 clone PgR-636. Normal human breast tissue was used as a positive control for both markers. Nuclear expression was quantified using histoscore, generated by multiplying the proportion of positive tumour nuclei (0–100%) by the intensity of nuclear staining (0–3) to produce weighted scores from 0 to 300^52^ (Supplementary Fig. S1). Two independent observers (CSH, RLH) scored digital images of stained whole slides, demonstrating excellent agreement (rho = 0.96 for PR, rho = 0.93 for ER). The median absolute difference in histoscore between observers was 10 and 20 for PR and ER, respectively. The final histoscore was calculated as the mean score of the two observers.

### Identification of invasive patterns and desmoplasia

During review, the presence of desmoplasia, macropapillary (MaP) stromal invasion and micropapillary (MiP) stromal invasion was recorded^53^. H&E slides from all available tumour specimens for each patient were examined for each histopathological feature. The presence of MiP was defined as invasion of stroma by papillary structures that did not contain fibrovascular cores (Fig. 1A,B). LGSOC cases were classified as having MaP if stained slides showed definitive stromal invasion by papillary structures containing fibrovascular cores^53^ (Fig. 1C–F). The presence or absence of each pattern of invasion was recorded for each case. The presence of desmoplasia was defined as reactive growth of fibrotic stromal (loose elongated stromal cells) in proximity to invasive tumour (Fig. 1F).

### Genomic profiling

Whole exome sequencing data were available for the cohort from the previous genomic profiling study^50^. Tumour DNA underwent whole exome sequencing to a median per-sample on-target depth of 64× (samples with < 30× coverage were excluded from analysis) using the Illumina TruSeq Exome Library Prep Kit and the Illumina NextSeq 550 platform. Reads were aligned to GRCh38 and variants were called using a majority vote system from three separate variant callers (Freebayes, Mutect2, VarDict) within the bcbio nextgen workflow, then filtered to exclude non-functional variants as described previously^50^. For full details, see ref^50,51^.

Genome-wide CN data were derived from aligned BAM files using the CopywriteR R package to calculate relative CN estimates at 30kB genomic intervals^54^. Median relative log2 CN ratios of intervals spanning each chromosome arm were calculated; a median of 0.25 and -0.25 were used as thresholds for chromosome arm-level gains and losses, respectively.

### Survival analysis

Survival analysis was performed using Cox proportional hazards models, with effect sizes reported as hazard ratios (HR) and corresponding 95% confidence intervals (95% CI). The overall survival, progression-free survival and disease-specific survival event rates were 60% (38 of 63), 52% (32 of 61 evaluable cases) and 38% (21 of 55 evaluable cases). The estimated power to detect a strong effect size (HR 0.3) was 86%, assuming 1:1 allocation to two groups and an event rate of 50%.

### Statistical analysis

Continuous variables were compared using the Mann-Whitney U test. Comparisons of categorical data were performed using the Chi-squared test for larger sample sizes, and Barnard’s test for smaller sample sizes. Multiple testing correction was performed using the Bonferroni method to produce multiplicity-adjusted P-values (P-adj). All analyses were performed using R version 4.0.3.

## Supplementary Information

Supplementary Information.

## Data Availability

Data on the histopathological features of each case are available in Supplementary Table S4 of this manuscript. All other data generated and/or analysed during this study are available from the corresponding author upon reasonable request, subject to requests falling within our local ethics framework.
