# Supplementary material for: Distinct histopathological features are associated with molecular subtypes and outcome in low grade serous ovarian carcinoma
Source: Sci Rep. 2023 May 11;13:7681. doi: 10.1038/s41598-023-34627-5 (PMC10175560; doi:10.1038/s41598-023-34627-5)
Supplement: Supplementary file 1 — Supplementary Information. [file 41598_2023_34627_MOESM1_ESM.pdf]

SUPPLEMENT

Distinct histopathological features are associated with molecular subtypes and outcome in low grade serous ovarian carcinoma

Robert L Hollis[1a*], John P Thomson[1a], Juliette van Baal[2a], Narthana Ilenkovan[1,3], Michael Churchman[1], Koen van de Vijver[2], Frederike Dijk[4], Alison M Meynert[5], Clare Bartos[1], Tzyvia Rye[1], Ian Croy[1], Patricia Diana[1], Mignon van Gent[4], Helen Creedon[6], Rachel Nirsimloo[6], Christianne Lok[2b], Charlie Gourley[1,b] and C. Simon Herrington[1,b*]

[1]Nicola Murray Centre for Ovarian Cancer Research, Cancer Research UK Scotland Centre, Institute of Genetics and Cancer, University of Edinburgh, Edinburgh, UK

[2]Department of Gynaecologic Oncology and Department of Pathology, The Netherlands Cancer Institute, Antoni van Leeuwenhoek, Amsterdam, The Netherlands

[3]Cancer Research UK Scotland Centre, Beatson Institute for Cancer Research, Glasgow, UK

[4]Department of Gynaecologic Oncology and Department of Pathology, Amsterdam University Medical Centres, Amsterdam, The Netherlands

[5]MRC Human Genetics Unit, Institute of Genetics and Cancer, University of Edinburgh, Edinburgh, UK

[6]Edinburgh Cancer Centre, Western General Hospital, NHS Lothian, Edinburgh, UK.

[a,b]Authors contributed equally

***Corresponding Author(s):** Dr Robb Hollis (robb.hollis@ed.ac.uk) and Prof C. Simon Herrington (simon.herrington@ed.ac.uk). Nicola Murray Centre for Ovarian Cancer Research, Cancer Research UK Scotland Centre, IGC, University of Edinburgh, Crewe Road South, Edinburgh, EH4 2XU, UK

Supplementary tables

Table S1. Multivariable analysis of micropapillary (MiP) invasion pattern and disease specific survival

		Hazard ratio	95% interval	P-value
**MiP**	Present	reference	-	-
	Absent	0.29	0.10-0.88	0.0293
**FIGO stage**	I-II	reference	-	-
	III-IV	1.76	0.34-9.01	0.4959
**Residual disease**	No visible residual disease	0.22	0.06-0.75	0.0154
	Macroscopic residual disease	reference	-	-

Table S2. Multivariable analysis of desmoplasia and disease specific survival

		Hazard ratio	95% interval	P-value
**Desmoplasia**	Present	reference	-	-
	Absent	0.25	0.06-1.02	0.0535
**FIGO stage at diagnosis**	I-II	reference	-	-
	III-IV	0.63	0.10-4.20	0.6373
**Residual disease status**	No visible residual disease	0.21	0.06-0.77	0.0186
	Macroscopic residual disease	reference	-	-

Table S3. Multivariable analysis of progesterone receptor (PR) expression and disease specific survival

		Hazard ratio	95% interval	P-value
**PR**	Histoscore <50	reference	-	-
	Histoscore ≥50	0.33	0.12-0.93	0.0364
**FIGO stage at diagnosis**	I-II	reference	-	-
	III-IV	1.07	0.21-5.33	0.9355
**Residual disease status**	No visible residual disease	0.24	0.07-0.82	0.0223
	Macroscopic residual disease	reference	-	-

Table S4. Presence or absence of macropapillary (MaP) invasion, micropapillary (MiP) invasion and desmoplasia across 63 cases

Case	MaP	MiP	Desmoplastic
**C1**	FALSE	TRUE	FALSE
**C2**	FALSE	TRUE	FALSE
**C3**	FALSE	TRUE	TRUE
**C4**	TRUE	FALSE	FALSE
**C5**	TRUE	TRUE	TRUE
**C6**	TRUE	FALSE	TRUE
**C7**	FALSE	TRUE	TRUE
**C8**	FALSE	FALSE	FALSE
**C9**	TRUE	FALSE	TRUE
**C10**	TRUE	FALSE	TRUE
**C11**	FALSE	TRUE	TRUE
**C12**	FALSE	TRUE	FALSE
**C13**	FALSE	TRUE	FALSE
**C14**	FALSE	TRUE	FALSE
**C15**	TRUE	TRUE	TRUE
**C16**	FALSE	TRUE	TRUE
**C17**	FALSE	TRUE	FALSE
**C18**	TRUE	FALSE	TRUE
**C19**	FALSE	TRUE	TRUE
**C20**	TRUE	TRUE	TRUE
**C21**	FALSE	TRUE	TRUE
**C22**	TRUE	FALSE	TRUE
**C23**	FALSE	TRUE	FALSE
**C24**	FALSE	TRUE	TRUE
**C25**	FALSE	TRUE	FALSE
**C26**	TRUE	FALSE	TRUE
**C27**	TRUE	FALSE	TRUE
**C28**	TRUE	FALSE	FALSE
**C29**	FALSE	TRUE	TRUE
**C30**	TRUE	FALSE	FALSE
**C31**	FALSE	TRUE	TRUE
**C32**	FALSE	TRUE	FALSE
**C33**	FALSE	TRUE	TRUE
**C34**	FALSE	TRUE	TRUE
**C35**	TRUE	FALSE	FALSE
**C36**	FALSE	TRUE	FALSE
**C37**	FALSE	TRUE	TRUE
**C38**	TRUE	FALSE	TRUE
**C39**	TRUE	FALSE	TRUE
**C40**	TRUE	FALSE	TRUE
**C41**	FALSE	TRUE	TRUE
**C42**	TRUE	FALSE	TRUE

**C43**	FALSE	FALSE	FALSE
**C44**	FALSE	TRUE	TRUE
**C45**	FALSE	TRUE	TRUE
**C46**	FALSE	TRUE	TRUE
**C47**	TRUE	FALSE	TRUE
**C48**	FALSE	TRUE	TRUE
**C49**	FALSE	TRUE	TRUE
**C50**	TRUE	FALSE	TRUE
**C51**	FALSE	TRUE	FALSE
**C52**	TRUE	FALSE	TRUE
**C53**	FALSE	FALSE	FALSE
**C54**	FALSE	FALSE	FALSE
**C55**	FALSE	TRUE	FALSE
**C56**	FALSE	TRUE	TRUE
**C57**	FALSE	TRUE	TRUE
**C58**	FALSE	TRUE	TRUE
**C59**	FALSE	TRUE	TRUE
**C60**	FALSE	FALSE	FALSE
**C61**	TRUE	FALSE	TRUE
**C62**	FALSE	TRUE	FALSE
**C63**	TRUE	FALSE	FALSE

Supplementary figures

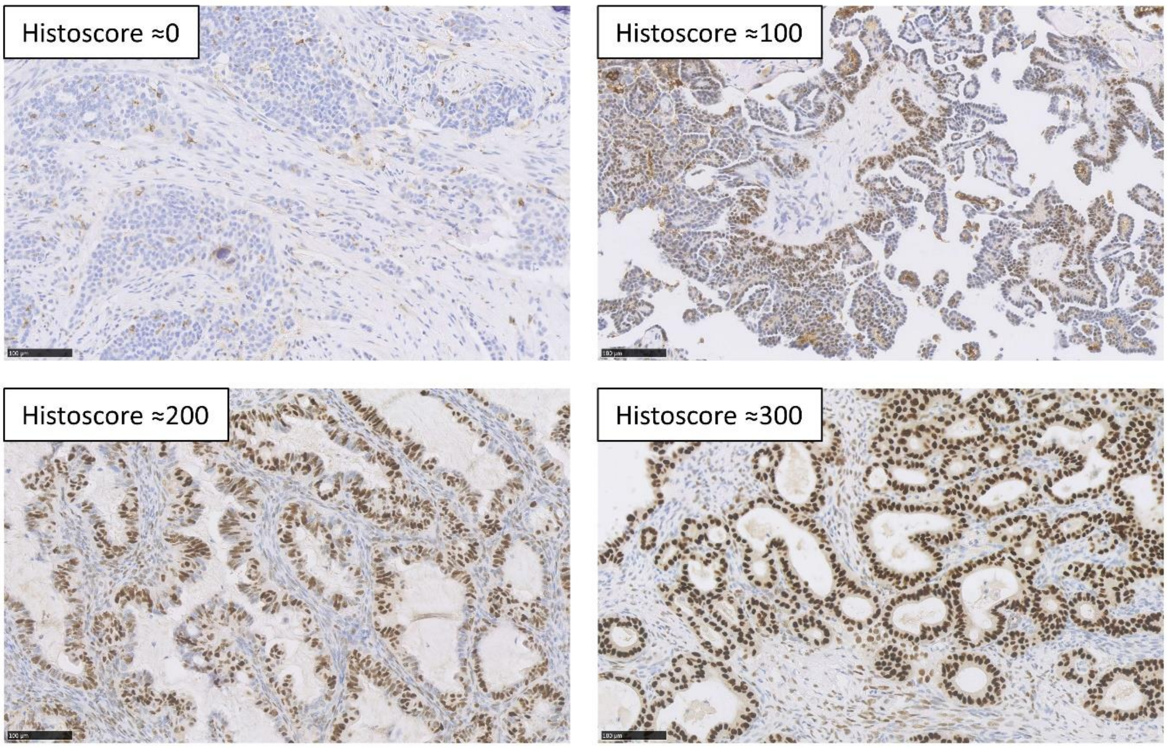

Figure S1. Example histoscoring to determine intensity of oestrogen and progesterone receptor expression by immunohistochemistry. Images taken at 20X magnification.

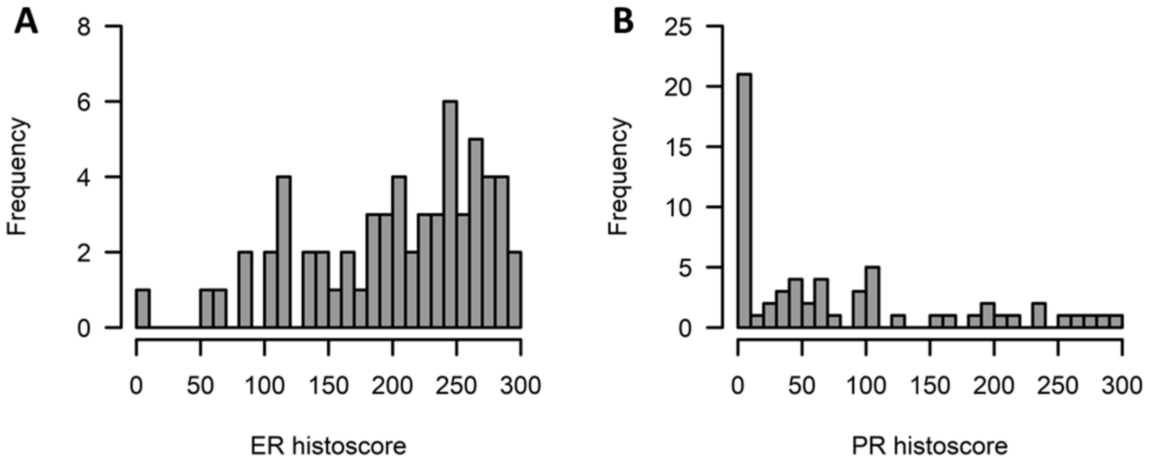

Figure S2. Histograms of hormone receptor expression, as determined by histoscore, across LGSOC cases. (A) Oestrogen receptor (ER) expression (n=61 evaluable cases). (B) Progesterone receptor (PR) expression (n=61 evaluable cases).

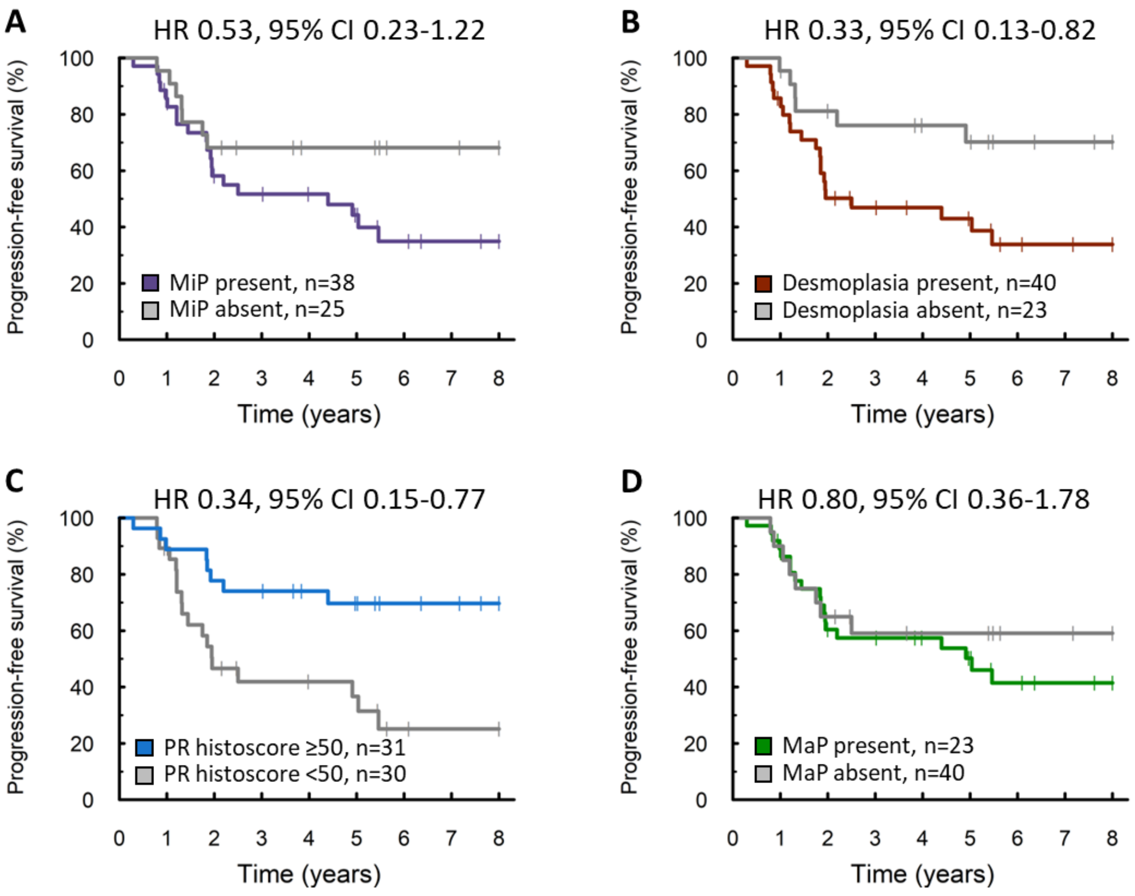

Figure S3. Univariable analysis of progression-free survival (PFS). A, PFS of cases with and without the micropapillary (MiP) pattern of stromal invasion. Labelled hazard ratio represent comparison of cases without MiP versus cases with MiP stromal invasion. B, PFS of cases with and without desmoplasia. Labelled hazard ratio represent comparison of cases without desmoplasia identified versus desmoplastic cases. C, DSS of cases classified by progesterone receptor (PR) expression levels. Labelled hazard ratio represent comparison of cases with PR histoscore ≥50 (the median) versus PR histoscore <50. D, DSS of cases with and without the macropapillary (MaP) pattern of stromal invasion. Labelled hazard ratio represent comparison of cases with MaP versus cases without MaP stromal invasion.

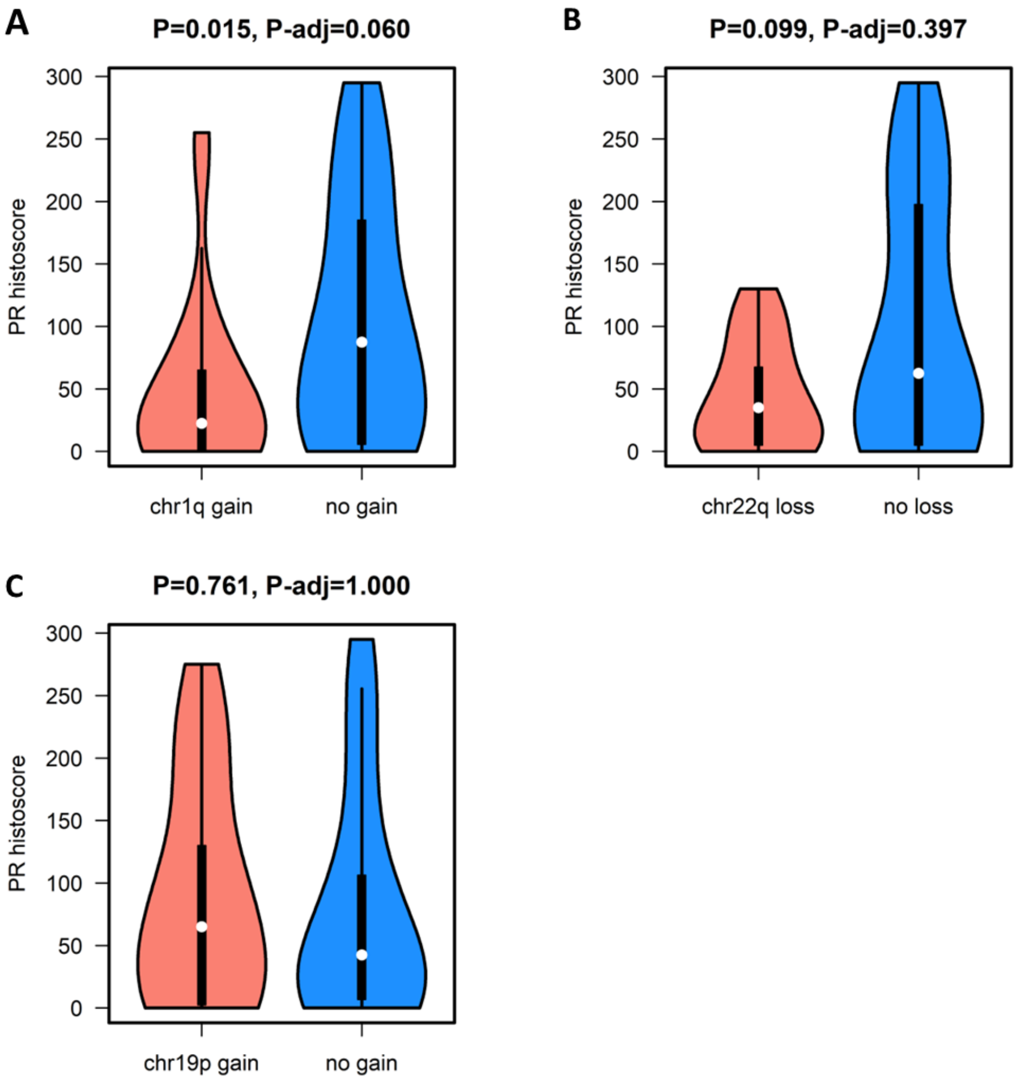

Figure S4. Comparison of progesterone receptor expression between LGSOC groups defined by (A) chr1q gain, (B) chr22q loss and (C) chr19p gain. P values adjusted (P-adj) using the Bonferroni method for n=4 comparisons (PR expression versus chr1p loss, chr22q loss, chr1q gain and chr19p gain).
